# Quantitative IVIM parameters evaluating perfusion changes in brain parenchyma in patients newly diagnosed with acute leukemia: Compared with healthy participants

**DOI:** 10.3389/fneur.2023.1093003

**Published:** 2023-02-02

**Authors:** Jianing Cui, Jing Zheng, Weiran Niu, Wenjin Bian, Jun Wang, Jinliang Niu

**Affiliations:** ^1^Medical Imaging Department, Shanxi Medical University, Taiyuan, Shanxi, China; ^2^School of Basic Medical Sciences, Shanxi Medical University, Taiyuan, Shanxi, China; ^3^Department of Mental Health, Shanxi Medical University, Taiyuan, Shanxi, China; ^4^Department of Radiology, Second Hospital, Shanxi Medical University, Taiyuan, Shanxi, China

**Keywords:** gray matter, white matter, IVIM, acute leukemia, cerebral blood perfusion, white blood cell counts

## Abstract

**Purpose:**

To study the value of quantitative IVIM parameters in evaluating cerebral blood perfusion changes in patients newly diagnosed with acute leukemia (AL) by comparing them with healthy participants.

**Materials and methods:**

This prospective study consecutively recruited 49 participants with newly diagnosed AL and 40 normal controls between July 2020 and September 2022. All participants underwent an MRI of the brain using an axial T_1_-weighted and an IVIM sequence. The IVIM parameters (water diffusion coefficient, sADC, pseudoperfusion fraction, *f*; diffusion coefficient, *D*, pseudodiffusion coefficient, D^*^, and perfusion-diffusion ratio, PDR) and peripheral white blood cell (WBC) counts were obtained. An unpaired *t-*test or the Mann–Whitney U-test was performed to compare the differences in gray matter (GM) and white matter (WM) of healthy participants and AL patients and the differences in IVIM parameters between healthy participants and patients with AL. In addition, multivariate (logistic regression) analyses were used to identify independent predictors and then, the receiver operating characteristic curve (ROC) analyses were performed.

**Results:**

40 healthy participants and 49 patients with newly diagnosed AL were evaluated. In healthy participants, sADC, PDR, *D* and *f* values of GM were significantly higher than those of WM (*t* = 5.844, *t* = 3.838, *t* = 7.711, *z* = −2.184, respectively, all *P* < 0.05). In AL patients, the *D, f* and sADC values of GM were significantly higher than those of WM (*t* = 3.450, *t* = 6.262, *t* = 4.053, respectively, all *P* < 0.05). The sADC and *f* value from AL patients were significantly lower than those from healthy participants in GM (*z* = −2.537, *P* = 0.011; and *z* = −2.583, *P* = 0.010, respectively) and WM (*z* = −2.969, *P* = 0.003; *z* = −2.923, *P* = 0.003, respectively). The WBC counts of AL patients were significantly higher than those of healthy participants (*t* = 3.147, *P* = 0.002). Multivariate analyses showed that the *f* values of GM and WM were independent predictors of AL (*P* = 0.030, and 0.010, respectively), with the optimal cut-off value at 7.08% (AUC ROC curve: 0.661, specificity: 11.4%, sensitivity: 98%) and 13.77% (AUC ROC curve: 0.682, specificity: 79.5%, sensitivity: 59.2%).

**Conclusion:**

The IVIM parameters of brain parenchyma in patients newly diagnosed with AL differed from those of the healthy participants. The changes of cerebral blood flow perfusion are expected to provide new ideas for studying central nervous system infiltration in AL.

## 1. Introduction

Both gray matter (GM) and white matter (WM) in the brain are important components of the brain parenchyma of the central nervous system (CNS). GM is located on the brain's surface, which is rich in neurons and has a higher capillary density (1). WM is located beneath the GM, which is mainly composed of myelinated nerve axons, myelin-producing glial cells, and lower blood supply (2–4). Most brain diseases change the perfusion of the brain parenchyma (5, 6). Thus, studying the microcirculation feature of GM and WM could help us further understand the development of disease and formulate corresponding treatment measures in time (7).

Acute leukemia (AL) is a malignant clonal disease of hematopoietic stem cells in marrow (8, 9), which can infiltrate extramedullary organs (the liver, the spleen, the renal system, and so on) and the central nervous system (10–12). Some studies suggested that leukemia cells can damage the blood–brain barrier (BBB) and spread leukemic cells across the leptomeninges to the adjacent brain parenchyma, cross the GM, pass the junctional area, and reach the WM (12, 13). At the same time, ~5–30% of patients with AL have increased peripheral white blood cell (WBC) counts (8, 9). Hyperleukocytosis results in leukostasis, which affects microcirculation perfusion (14, 15). Thus, we hypothesize that AL patients probably occur perfusion changes of brain parenchyma.

Currently, imaging methods commonly used to study cerebral blood perfusion include dynamic contrast-enhanced magnetic resonance imaging (DCE-MRI) (16), arterial spin labeling (ASL) (17), intravoxel incoherent motion (IVIM) (18), and so on. DCE-MRI is widely used to assess blood perfusion in strokes and brain tumors. Previous studies used DCE-MRI to prove that the perfusion parameters of GM and WM are different in healthy participants, such as cerebral blood volume (CBV) was significantly higher in GM than WM, with a mean pair-wise GM/WM CBV ratio of 1.9 (16). However, the application of exogenous contrast agents is limited. It has also shown that ASL can reflect blood perfusion. In the central nervous system, ASL is often used to evaluate perfusion in ischemic brain diseases and hypoxic brain injury. A previous study showed that the areas of increased diffusion signals showed increased ASL signals (19). Tiwari et al. conducted a study on rats, and the result showed that the CBF in the ASL stroke model could detect mild BBB leakage in the early stage compared with the standard DCE-MRI (20). However, ASL is very sensitive to motion and requires the patient to be immobile. It takes a long time to scan, which is intolerable for some AL patients (17). The ASL parameter is single, and only CBF reflects perfusion (21, 22).

IVIM is a diffusion-weighted imaging method that uses multiple b-values and a double-exponential signal model that enables quantitative parameters to reflect tissue microcapillary perfusion and tissue diffusivity, respectively (18, 19). IVIM does not require the injection of an exogenous contrast agent, has no nephrotoxicity, and can assess cerebral blood flow perfusion noninvasively (23, 24). The only IVIM parameters earlier were the water diffusion coefficient (sADC), the true diffusion coefficient (*D*), the pseudodiffusion coefficient (D^*^), and the perfusion fraction (*f*), which could evaluate the perfusion and water diffusion characteristics (24, 25). Recently, to improve the validity of IVIM parameters to characterize specific tissues, attention has been paid to a new IVIM parameter, the perfusion-diffusion ratio (PDR), which expresses the relationship between the rate of S(b) signal decline induced by IVIM and that induced by diffusion (26). IVIM can also be used to assess perfusion in Alzheimer's disease, cerebral small vessel disease, and tumors (21, 24, 25). For example, Wong et al. performed the first study using IVIM in cerebral small vessel disease patients, and they observed an increase in perfusion fraction (21). IVIM could also discriminate between high-grade and low-grade diffuse gliomas (25).

This study aimed to investigate the value of IVIM quantitative parameters in evaluating cerebral blood perfusion in patients with AL and to further compare the differences between patients with acute lymphoblastic leukemia (ALL) and acute myeloid leukemia (AML). It was also discussed that leukostasis caused by leukocytosis might be one of its causes.

## 2. Materials and methods

### 2.1. Patients

This study was approved by the Local Ethics Committee, and written informed consent was obtained from all patients and healthy participants. From July 2020 to September 2022, 40 healthy participants and 49 patients newly diagnosed with AL confirmed by the WHO classification of hematopoietic tissue were prospectively enrolled in this study and underwent an MRI of the brain and routine blood examination. The inclusion criteria were as follows: (1) absence of prior chemotherapy or radiotherapy and (2) stable vital signs for an MRI examination and no contraindications to an MRI examination. The exclusion criteria included patients with other diseases in the CNS and poor image quality in IVIM. Healthy participants matching in age and sex with AL patients and without history of central nervous system disease were recruited as the control group.

### 2.2. MRI parameters

MR examinations were performed with a 3.0T scanner (GE Healthcare, 750W, Milwaukee, WI) using a multichannel phased-array head coil. The MR sequences consisted of anaxial T_1_-weighted (repetition time [TR] = 3,569.5 msec; echo time [TE] = 24.96 msec; section thickness = 6.0 mm; no gap; number of excitations [NEX] = 1; field of view [FOV] = 24 × 24 cm; matrix = 320 × 256; acquisition time = 2 min and 5 s). The IVIM sequence was based on the standard diffusion-weighted single-shot spin-echo planar imaging with 11 b-values (0, 10, 20, 30, 40, 50, 100, 200, 400, 800, and 1,000 s/mm^2^); TR = 7,500 msec, TE = 78.40 msec, slice thickness = 6.0 mm; no gap; NEX = 4; FOV = 24 × 24 cm; matrix = 128 × 128; acquisition time = 5 min and 53 s. Fat suppression based on a spectral–spatial excitation pulse was used in IVIM.

### 2.3. Blood routine examination

WBC counts were recorded for all healthy participants and patients newly diagnosed with AL.

### 2.4. Image analysis

All data were transferred to an Advantage Windows Workstation 4.6 (GE Healthcare) for processing the IVIM parametric maps (sADC, *D*, D^*^, and *f*), which were derived from a biexponential fitting model, combining T1WI images with IVIM images in the enlarged T1WI images, and rectangular regions of interest (ROIs) ~8–11 mm^2^ were placed according to the size of the WM and GM of the brain in T1WI images in the frontal, parietal, temporal and occipital lobes of the brain (27). The selection of ROIs is shown in **Figure 2**. All ROIs were selected with bilateral symmetry, and the selected level was the same for each patient. All the plotted ROIs were discussed and confirmed by two experienced radiologists who were blinded to clinical information.

The IVIM model is a standard two-compartment model of diffusion, which is described by a biexponential equation:


(1) $$\begin{array}{l} \frac{S(b)}{S0}=f\exp[-bD^{*}]+(1-f)\exp\;[-bD] \end{array}$$

18.

Other parameters of IVIM were calculated using the following formula:

*f* (perfusion fraction): *f* = 1 – S(int)/S(0) (28), D^*^(pseudodiffusion coefficient): D^*^= lv/6 (18), *D* (pure diffusion coefficient): S(b)/S(0) = exp(–b*D*) (29).

The PDR can be calculated as follows:


(2) $$\begin{array}{l} PDR=\frac{f}{1-f}*\frac{D^{*}}{D}\; \end{array}$$

26.

### 2.5. Statistical analysis

An unpaired *t-*test or the Mann–Whitney U-test was performed to compare the differences in GM and WM of healthy participants and patients newly diagnosed with AL and the differences in IVIM parameters and WBC count between healthy participants and patients with AL. A significance level of *P* of <0.05 was considered statistically significant. Variables with a *P*-value of <0.05 in univariate analyses were included as covariates in multivariate (logistic regression) analyses. Predicting performance was assessed using the AUC of the ROC curve. The statistical analyses were performed with SPSS software (v. 25.0, IBM, Armonk, NY).

## 3. Results

### 3.1. Study participants

**Figure 1:**
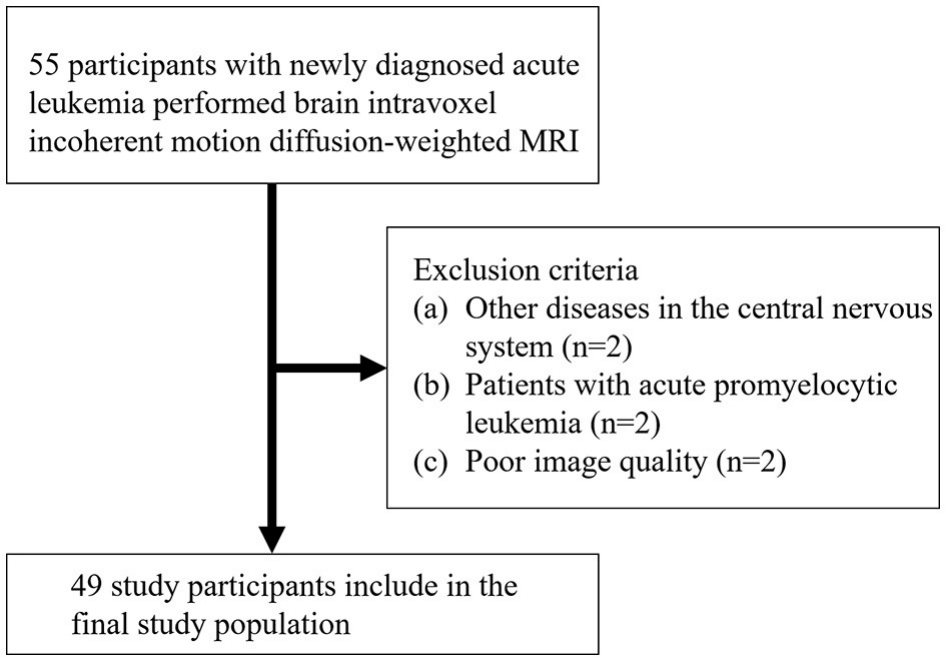
Participant inclusion and exclusion flowchart.

A total of 55 AL patients underwent MRI. Figure 1 shows the initial number of participants with AL, the number of participants excluded, and the final study sample. The final study sample consisted of 49 patients with AL (mean age, 47.7 years ±17.1 [standard deviation]; age range, 16–8 years): 26 men and 23 women. Of these, 12 were patients with ALL and 37 were patients with AML.

### 3.2. Comparison of IVIM parameters between GM and WM

In 40 healthy participants, sADC and PDR and the *D* and *f* values of GM were significantly higher than those of WM (*t* = 5.844, *P* < 0.001 and *t* = 3.838, *P* < 0.001, *t* = 7.711, *P* < 0.001 and *z* = −2.184, *P* = 0.029 respectively, Table 1); the D^*^ value of GM were significantly lower than that of WM (*z* = −2.284, *P* = 0.022). The numerical values are shown in Table 1.

**Table 1:** IVIM parameters of healthy participants and AL patients.

	**Healthy participants**		**AL patients**		***P*** **-value**			
	**GM**	**WM**	**GM**	**WM**	*P*_a_	*P*_b_	*P*_c_	*P*_d_
sADC(× 10^−3^mm^2^/s)	1.13 ± 0.25	0.88 ± 0.08	0.98 ± 0.22	0.87 ± 0.17	*t =* 5.844 *P* < 0.001	*t =* 3.450 *P =* 0.001	*z =* −2.537 *P =* 0.011	*z =* −2.969 *P =* 0.003
*D* (× 10^−3^mm^2^/s)	0.96 ± 0.15	0.82 ± 0.16	1.01 ± 0.16	0.82 ± 0.14	*t =* 3.838 *P* < 0.001	*t =* 6.262 *P* < 0.001		
D^*^ (× 10^−3^mm^2^/s)	26.12 ± 22.09	37.43 ± 24.98	29.42 ± 14.61	33.23 ± 18.72	*z =* −2.284 *P =* 0.022			
*f* (%)	26.81 ± 11.94	11.62 ± 3.52	21.41 ± 7.83	10.63 ± 6.42	*t =* 7.711 *P* < 0.001	*t =* 4.053 *P* < 0.001	*z =* −2.583 *P =* 0.010	*z =* −2.923 *P =* 0.003
PDR	8.20 ± 5.64	6.19 ± 5.92	7.73 ± 4.73	8.347 ± 6.14	*z =* −2.184 *P =* 0.029			

*P*_a_, *P* value of GM and WM IVIM parameters in healthy participants.

*P*_b_, *P* value of GM and WM IVIM parameters in AL patients.

*P*_c_, *P* value of GM IVIM parameters in healthy participants and AL patients.

*P*_d_, *P* value of WM IVIM parameters in healthy participants and AL patients.

In 49 AL patients, sADC as well as the *D* and *f* values of GM were significantly higher than that of WM (*t* = 3.450, *P* = 0.001, *t* = 6.262, *P* < 0.001 and *t* = 4.053, *P* < 0.001 respectively, Table 1).

### 3.3. Comparison of IVIM parameters and WBC counts between healthy participants and patients newly diagnosed with AL

**Figure 2:**
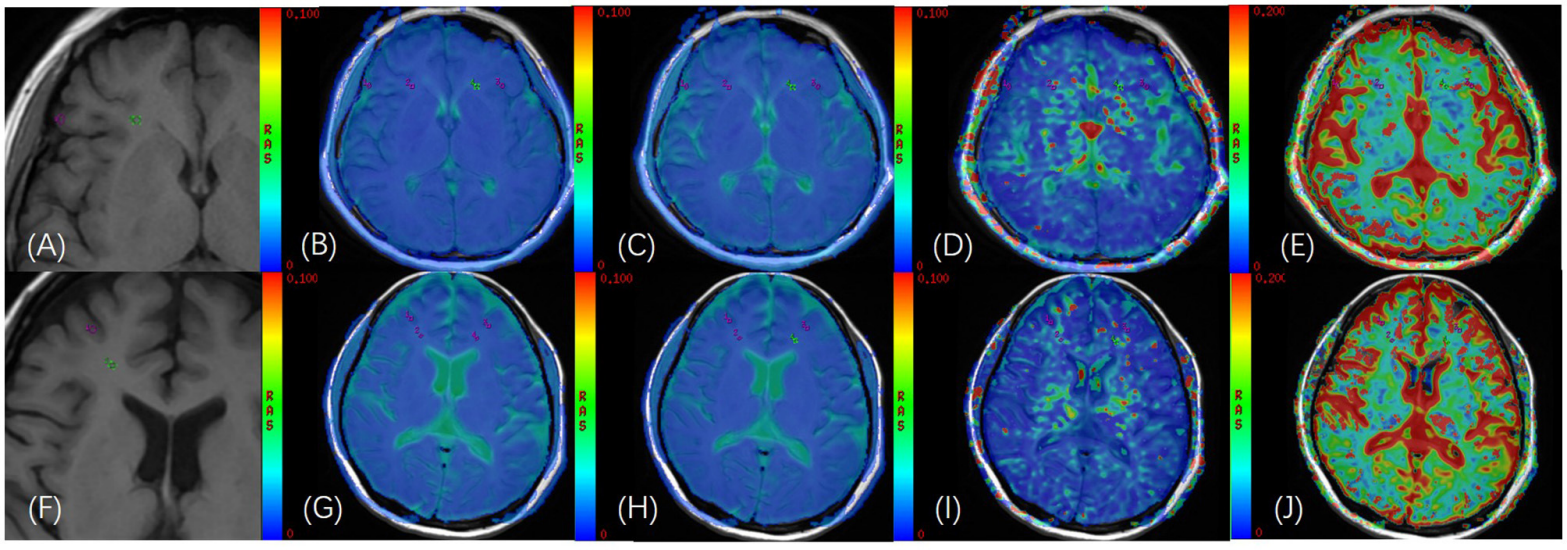
**(A)** T_1_WI images of a 40-year-old female healthy participant; **(B–E)** GM and WM of bilateral frontal lobes were delineated on the fusion image of axial IVIM functional image [apparent diffusion coecient (sADC), *D*, D^*^, and *f*, respectively] and axial T_1_WI image, and the ROI area was 11 mm^2^; **(F)** T_1_WI images of a 50-year-old female patient with AL; **(G–J)** GM and WM of bilateral frontal lobes were delineated on the fusion image of axial IVIM functional image [apparent diffusion coecient (sADC), *D*, D^*^, and *f*, respectively] and axial T_1_WI image, and the ROI area was 11 mm^2^.

In 40 healthy participants and 49 patients with AL, in GM, sADC and the *f* value from AL were significantly lower than those from healthy participants (*z* = −2.537, *P* = 0.011, *z* = −2.583, *P* = 0.010, respectively, Table 1) in GM; sADC and the *f* value of AL were significantly lower than those from healthy participants (*z* = −2.969, *P* = 0.003, *z* = −2.923, *P* = 0.003, respectively, Table 1) in WM; the WBC count of the 49 AL patients was significantly higher than that of 40 healthy participants (*t* = 3.147, *P* = 0.002); IVIM parametric maps of sADC, *D*, D^*^ and *f* in a healthy participant and a patient with AL are illustrated in Figure 2.

**Figure 3:**
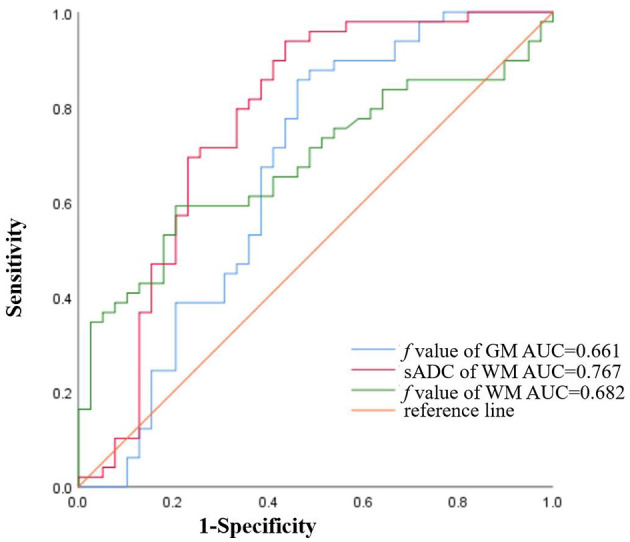
The ROC curves of sADC of WM (AUC = 0.767), the *f* value of GM (AUC = 0.661), and the *f* value of WM (AUC = 0.682) for diagnosing AL.

Multivariate analyses showed that the *f* value of GM and WM were independent predictors of AL (*P* = 0.03, and *P* = 0.01, respectively). The numerical values are shown in Table 2. In the ROC analysis, the *f* value of GM (cutoff of 7.08%) had a sensitivity of 98%, a specificity of 11.4%, and an AUC of 0.661 (*P* = 0.010; 95% CI: 0.538–0.784) (Figure 3); and sADC of WM (cutoff of 0.79 × 10^−3^mm^2^/s) demonstrated a sensitivity of 94%, a specificity of 56.4%, and an AUC of 0.767 (*P* < 0.001; 95% CI: 0.658–0.876) (Figure 3). The *f* value of WM (cutoff of 13.77%) achieved a sensitivity of 59.2%, a specificity of 79.5%, and an AUC of 0.682 (*P* = 0.003; 95% CI: 0.571–0.793) (Figure 3).

**Table 2:** Multivariate (logistic regression) analyses in healthy participants and patients with AL.

**Parameters**	**β**	**S.E**.	**Wald**	***P***
sADC of GM (10^−3^ mm^2^/s)	−1,403.90	1,099.38	1.63	0.21
sADC of WM (10^−3^ mm^2^/s)	−460.50	1,629.48	0.08	0.78
*f* value of GM (%)	5.863	2.77	4.49	0.03
*f* value of WM (%)	−15.31	5.91	6.7i	0.01
WBC (× 10^9^)	−0.06	0.03	3.57	0.06

### 3.4. Comparison of IVIM parameters in GM and WM between AML and ALL

Comparing 12 ALL patients with 37 AML patients, in GM, the D^*^ value of ALL was significantly lower than that of AML (*z* = −2.464, *P* = 0.014); in WM, the D^*^ value and PDR of ALL were significantly lower than that of AML (*z* = −2.395, *P* = 0.017 and *z* = −2.674, *P* = 0.008, respectively).

## 4. Discussion

It is of great significance to study cerebral blood perfusion. Most brain diseases change the perfusion of the brain parenchyma (5, 6). Leukemia is considered a metastatic systemic disease, including extramedullary infiltration and central nervous system leukemia (CNSL). It causes changes in the microcirculation of systemic organs, such as the liver, the spleen, the kidney, and so on (10–12). Thus, we suspect that the brain microcirculation may also change in AL. Studying the microcirculation feature in the brain could help us further understand the development of disease (7). IVIM is a noninvasive imaging method and has been used in the study of brain diseases. Our results indicate differences in cerebral blood perfusion between patients with AL and healthy participants.

In 40 healthy participants, sADC as well as the *D* and *f* values of GM were significantly higher than those of WM. This is consistent with the microcirculation of the blood supply to the brain. Histological study in the human brain showed that vascular density is higher in GM than in WM. Although the pathophysiologic meaning of IVIM parameters needs to be further investigated, the *f* and *D* values have been shown as imaging markers to separately assess vascular volume fraction of angiogenesis and cellularity in solid and hematologic tumors. At low b values, *f* and D^*^ can evaluate perfusion characteristics; at high b values, *D* can reflect water diffusion related to tissue cellularity (30). This approach has potential in the diagnosis of tumor lesions and in evaluating treatment responses in oncology. PDR, another parameter of IVIM, can be used to evaluate capillary permeability and has a certain value in the differentiation of primary liver solid space-occupying lesions (26). Although there are few studies on PDR, it might supplement future studies.

Similar results were found in the brains of patients with AL but not in PDR. The possible reason is that the PDR is obtained after the calculation of *D*, D^*^, and *f*, and the sensitivity is relatively low. This result shows that, although slightly different from healthy participants, the blood supply of GM and WM in the brain of patients with AL is relatively stable. Previous studies showed that GM should have 3–5 times higher perfusion than WM. Our study also showed that GM has higher blood flow than WM and that GM has more than two times as much blood flow as WM. The possible reason is that the IVIM parameters have different biological significance, which needs to be confirmed by pathology. In 40 healthy participants and 49 patients newly diagnosed with AL, for both GM and WM, sADC and the *f* value of AL were significantly lower than those of the healthy group. The possible reason is that patients with AL have a higher WBC count, resulting in a state of WBC stasis. Low sADC indicates possible diffusion limitation in the brain parenchyma, which might also be related to leukocyte stasis in the vessels of the CNS. The low *f* value may be related to the decrease of vascular content in the brain parenchyma, which is also most likely due to the stasis of WBCs. In our study, sADC of GM and WM did not show statistical significance in multivariate analysis. We speculate that the possible reason is that GM has more blood flow than WM and is less sensitive to changes in blood flow perfusion. After multivariate analysis, the ROC curve analysis was performed. In the ROC analysis, the *f* value of GM, sADC of WM, and the *f* value of WM all had cutoff values. This can further demonstrate the feasibility of this study.

Previous studies showed that CNSL is more likely to occur in patients with ALL than in patients with AML. However, it is unclear whether there is a difference in cerebral perfusion between AML and ALL. The results of our study show that D^*^ values and PDR are different in patients with ALL than in patients with AL. D^*^ can evaluate perfusion characteristics; CNSL may be related to the pathogenesis of patients with AL. This indicates differences in blood perfusion between patients with ALL and patients with AML, and the possible mechanism was the difference in the blood–brain barrier. The relevant mechanism will be further investigated in future studies.

There were some limitations in our study. First, the sample size was small. Further studies with more patients at different disease stages are needed to explore the changes in blood perfusion during the onset of the disease. Second, multimodal functional imaging (e.g., ASL, CEST) would help to comprehensively evaluate cerebral blood perfusion. Third, the study evaluated only changes in IVIM parameters in patients with AL, and the changes in IVIM parameters in patients with CNSL will be observed in the subsequent study to further explore the pathophysiological mechanism of CNSL.

In conclusion, we found that IVIM quantitative parameters of brain parenchyma in patients newly diagnosed with AL differed from those in healthy participants. Additionally, the D^*^ value was significantly lower in patients with ALL than that of patients with AML, which may indicate changes in cerebral blood perfusion. This is expected to provide new ideas for studying central nervous system infiltration in AL.

## Data availability statement

The raw data supporting the conclusions of this article will be made available by the authors, without undue reservation.

## Ethics statement

The studies involving human participants were reviewed and approved by Ethics Committee of the Second Hospital of Shanxi Medical University. The patients/participants provided their written informed consent to participate in this study. Written informed consent was obtained from the individual(s) for the publication of any potentially identifiable images or data included in this article.

## Author contributions

Guarantor of integrity of entire study: JN. Literature research: JC and WN. Clinical studies: JC, WN, and WB. Experimental studies: JZ. Statistical analysis: JC. Study concepts/study design or data acquisition or data analysis/interpretation, manuscript drafting or manuscript revision for important intellectual content, approval of final version of submitted manuscript, agrees to ensure any questions related to the work are appropriately resolved, and manuscript editing: All authors.
